# Alcohol Use as a Predictor of Risky Sexual Behaviour among Young Adults in the Western Cape Province of South Africa

**DOI:** 10.3390/ijerph20227053

**Published:** 2023-11-11

**Authors:** Cassandra Carels, Maria Florence, Sabirah Adams, Shazly Savahl

**Affiliations:** 1 Department of Psychology, University of the Western Cape, Cape Town 7535, South Africa; 2614996@myuwc.ac.za (C.C.); mflorence@uwc.ac.za (M.F.); 2 Language Development Group, Centre for Higher Education Development, University of Cape Town, Cape Town 7701, South Africa; sabirah.adams@uct.ac.za

**Keywords:** young adults, risky drinking, risky sexual behaviour, gender, employment status, low income communities, survey, AUDIT, street-intercept method, South Africa

## Abstract

The aim of this study was to investigate the relation between alcohol consumption and risky sexual behaviour (RSB). This study further aimed to examine whether alcohol use, gender, and employment status predicted RSB among young adults in the Cape Flats. A better understanding of these predictors could potentially lead to a more thorough comprehension of the relation between alcohol consumption and RSB among young adults within the South African context. The study employed a cross-sectional correlational survey design, with a sample of 1001 participants aged 18 to 25 (51% women), using the street-intercept method. Participants were selected using purposive sampling, with age and geographical location employed as inclusion criteria. We used the Self-Report Risky Sexual Behaviours Scale (SRSBS) and the Alcohol Use Disorder Identification Test (AUDIT), which were cognitively tested and modified, in English and Afrikaans. The data were analysed via multivariate multiple regression analysis in Stata. The key findings indicate that alcohol consumption is an important predictor of RSB. Alcohol consumption accounted for 23.22% of the variation in RSB. We also found that alcohol consumption and gender were significant (*p* < 0.1) predictors of RSB, but not employment status (*p* > 0.01). Being a woman decreased RSB. The model indicates that alcohol use and gender explain 18.41% of the variance in RSB. This study provides support for the growing body of research evidence that has established a significant link between alcohol consumption and RSB, highlighting the need for longitudinal research to determine patterns of risky drinking in the general population.

## 1. Introduction

Harmful consumption of alcohol is one of the leading global risk factors with a direct impact on many of the United Nations (UN) Sustainable Development Goals (SDGs) related to health [1,2]. Gender, age, health status, national economic prosperity, lifestyle choices, religion, and cultural norms influence alcohol consumption. Binge drinking is the dominant term used in the alcohol literature to describe people with excessive (i.e., contributing to intoxication) but episodic alcohol consumption [3]. Drinking alcohol continues to be a significant barrier to achieving 13 of the 17 Sustainable Development Goals (SDGs) by 2030, including SDG 10, which calls for the reduction of inequalities, the elimination of poverty, gender equality, good jobs and economic growth. Additionally, drinking fuels inequality within and between nations [4] by impeding progress towards SDG 10, which calls for less inequality. The association between alcohol consumption, risky sexual conduct, and HIV/AIDS is well established [5,6]. However, the literature recognises alcohol consumption and risky sexual behaviour (RSB) as two distinct variables that interact at a specific point that varies according to the features of the particular drinker and the sexual situation. Risky sexual behaviour (RSB) is defined in this article as having multiple sexual partners, having unprotected sexual encounters, concurrent sexual partners, transactional sex and physical and sexual violence [7,8,9,10].

Alcohol was used as a tool for land expropriation and removal during colonialism, which has a long history in South Africa and contributes to the country’s problematic alcohol use. Alcohol use played a substantial role in the operation of colonialism and was a crucial element of the oppressive practices in South Africa under apartheid. For example, farm workers were denied monetary compensation but instead remunerated with poor-quality alcohol [11]. Thus, alcohol misuse became embedded in the daily lives of farmworkers and informal workers [12]. The evolution of alcoholism in South Africa from colonial times to the present can be better understood in light of the apartheid laws, which shaped the country’s social and political landscape for nearly half a century. Apartheid was a form of structural violence and dominance of one group over another, based on a system of institutionalised racism, legal disenfranchisement, and overt discrimination. The effects of apartheid are experienced as a generational trauma that significantly motivates a variety of undesirable behaviours, including drug and alcohol abuse [13]. Alcohol use has thus become embedded in numerous social activities [7].

In addition to having the highest prevalence of HIV infections [8,9,14], South Africa also has the fifth-highest alcohol consumption prevalence worldwide [4]. Risky sexual behaviour (RSB) complicates prevention measures and is one of the main causes of the high incidence rates of HIV infections [15]. Heavy drinkers are more inclined to engage in hazardous sexual behaviours such as multiple partners, unprotected sex, multiple partners simultaneously, transactional sex, and sexual assault [8,9,10]. There has been an increase in South Africa’s consumption of high-alcohol-content alcoholic beverages in recent years [16,17,18]. Impaired judgment altered thinking processes, and a diminished sense of responsibility are all possible effects of alcohol use [8,19]. Therefore, the complex relation between alcohol use and RSB cannot be explained by a single mechanism [20,21]. The possible explanations include a cyclical and reciprocal effect of the environment influencing the individual and the individual influencing the environment; other explanations include the amount of casual sexual partners and the frequency of the use of condoms [21]. Previous research indicates that the connection between alcohol use and RSB is strengthened by the drinking context [22]. The drinking context is the physical (location, e.g., bar/club/restaurant vs. alone) and social environment (drinking in the presence of others vs. alone) of a community that influences alcohol consumption and the likelihood of problem drinking [23].

Young adults (15–24 years) often tend to engage in heavy drinking sessions (i.e., excessive alcohol consumption in one single session or one binge-drinking episode) [3,4]. Previous research [1] shows that, in several countries, including African countries, there is a consensus that current drinkers are likely to be heavy drinkers. Furthermore, due to increases in alcohol use during this age range, young adults aged 18 to 25 are most vulnerable to engaging in risky behaviour [24]. Men have a higher prevalence of heavy episodic drinking; however, women drink less than men [4]. Empirical studies have established a connection between youths’ RSB and their susceptibility to HIV and other sexually transmitted infections [25]. Young adults are vulnerable because they more often have multiple sexual partners (simultaneously or consecutively) [26] and are reluctant to use preventative measures, which include condom usage and regular testing [27].

A cross-sectional survey of secondary students was conducted in Kampala, Uganda [28]. The primary purpose of the study was to examine the attitudes of sexually active adolescents and young adults in secondary schools in Kampala, Uganda, regarding self-reported HIV testing and risky behaviour. Risky behaviour is associated with poor judgment, negligence, disputes with peers or people in authority, and a profoundly negative effect on society [29]. The main conclusions show that peer pressure and drug and alcohol use decreased students’ perceptions of the HIV risk, making them more vulnerable to HIV risk-related behaviours.

In-depth interviews and focus groups were used in a qualitative research study to investigate the risky behaviours of 18- to 24-year-old South African university students [30]. They paid special attention to comprehending the decline in condom use. Even though South Africa has one of the highest HIV/AIDS prevalence rates worldwide, students continue to participate in dangerous activities such as unprotected sex, multiple sexual partners, and alcohol use, despite being educated about the presumed risks involved with these practices. The study also revealed that male students had a greater tendency than female students to report RSB.

Survey-based research often excludes socially and economically disadvantaged populations, such as young adults who work or live on the streets [31]. This population is known to be vulnerable to a variety of health risks, including high rates of HIV risk behaviours associated with risky drug use. Despite the elevated incidence of alcohol use and alcohol use disorders among young adults [32], the relation between alcohol and sexual- and drug-related HIV risk behaviours has gained considerably less attention [31]. This population’s sexual- and drug-related risky behaviours have been the subject of extensive research [31]. Recent years have seen a dearth of intervention studies concerning RSB caused by alcohol and even fewer studies involving young adults [33]. There have also been studies among very similar populations, such as students at colleges and universities, particularly in the Global North [21]. The Global North is not a geographical region in the conventional sense; rather, it refers to the relative influence and wealth of countries in different parts of the world. The prosperous and powerful regions of North America, Europe, and Australia, among others, comprise the Global North [34]. However, the association between alcohol consumption and sexual behaviour in populations with low to moderate incomes has received insufficient attention [8].

An international quantitative study used the Self-Report Risky Sexual Behaviours Scale (SRSBS) to measure risky sex and included sexual trauma, pessimism, and depression to explain the variation in depression among students [35]. A higher SRSBS score indicates more reported instances of RSB, while a lower score indicates fewer instances. The measure was used in this study to look into any potential links between depression and people who report having more frequent RSB. They discovered that the variables accounted for a substantial portion of the variation in depression. Alcohol consumption was associated with higher incidences of depression in the study [35].

In low- and middle-income nations, the application and validity of the Alcohol Use Disorder Identification Test (AUDIT) cut-off scores have been studied [36]. The data were analysed using a narrative synthesis. Fifty-four studies were identified by the systematic review, the majority of which were carried out in India, Nigeria, and Brazil and employed AUDIT cut-off values distinct from those recommended by the World Health Organisation (WHO). The majority (*n* = 42) of these studies failed to perform psychometric evaluations of the AUDIT cut-off scores. In studies that reported psychometric results, a broad range of cut-off values performed well. The AUDIT is frequently used in low- to middle-income countries, where non-recommended cut-off values are accepted as appropriate [36]. The absence of psychometric information on the AUDIT cut-off scores utilised in the majority of studies was a significant finding. Despite the fact that many of these studies cited previous studies that validated these cut-off scores, studies conducted in the same social, economic, and cultural contexts were rarely cited. The WHO suggests cut-off scores to identify hazardous, harmful, and dependent alcohol use, but a large number of studies substituted terms like “low risk” or “binge drinking” for these. However, without using standardised terminology, it is not possible to know if the AUDIT cut-off scores in the various studies measure the same constructs, limiting their comparability.

In a previous study, South African researchers conducted a quantitative study to examine RSB and alcohol use among young adults in a low-income community in Cape Town, South Africa [37]. Students at the participating community’s secondary schools were specifically targeted by a participatory research model. A structured questionnaire consisting of the AUDIT and the SRSBS was administered using the street-intercept method. The main findings show a link between alcohol consumption and RSB (r = 0.48; *p* ≤ 0.01; *n* = 143). The study also shows that a significant proportion of participants were categorised as either harmful drinkers (men = 20.0%; women = 17.8%) or alcohol dependent (men = 54.3%; women = 47.9%). These findings demonstrate the lingering effects of the history and oppression and poverty that have fuelled intergenerational cycles of poverty, violence, and substance use in Cape Town and South Africa more generally.

Similarly, a systematic review examined the existing research on the variables that influence the association between alcohol use and RSB among young adults (18–24 years) [21]. The key finding was that personality traits, social determinants, and interpersonal factors are influential in the association between alcohol consumption and RSB. The systematic review also emphasised how the socio-political and historical context affects how modern people live their lives today. However, the literature was restricted to a specific region of North American, New Zealand and Australian youths’ patterns of risky behaviour [38,39,40], as it revealed that only a few studies focus on developing countries such as Kenya and South Africa [37,41,42,43]. Therefore, the authors recommended undertaking more studies using multiple methods to identify potential moderating variables affecting the relation between alcohol and RSB among young adults in South Africa.

A recent qualitative study in the South African context examined the factors that youth identify and understand as causes of alcohol use and subsequent RSB [7]. The sample consisted of young adults between the ages of 18 and 25 from low socioeconomic status (SES) communities in Cape Town, Western Cape, South Africa. Individual and social factors, each containing three themes, were identified as contributing to the association between alcohol use and RSB by young adults. The individual factors comprised intrapersonal influences, employment and educational attainment, and hope for the future, and the social factors comprised interpersonal influences, social and legislative impacts and influences. While these variables have been explored in the literature already, there remains a dearth of literature on young adults (excluding school-going and tertiary-going young people) in the South African context. These preliminary findings will make a valuable contribution to understanding the dynamics in this particular context and can point to how to explore it further in research.

### 1.1. Rationale for the Study

The link between alcohol use and RSB is well documented [8,37]; however, another study discovered that, despite being well-informed about the risk of unprotected sexual behaviour, young people continued to participate in RSB and alcohol consumption [30]. This has emerged as a major public health concern in South Africa and globally, particularly among young adults aged 18 to 25 [44,45,46]. Young adults in this age group are the most susceptible to engaging in risky drinking behaviours. Despite the literature from other countries and sub-Saharan Africa [47,48] supporting the global link between alcohol and HIV risk, there is a paucity of rigorous studies examining potential factors influencing this relation [49]. Personal influences, social determinants, and interpersonal factors have been highlighted by the research, contributing to a more detailed understanding of these factors [21]. Additional research is required at the most fundamental level to gain new insights, uncover new ideas, and/or increase understanding of the factors influencing the association between alcohol use and RSB in young adults [21]. A better understanding of these predictors could lead to a more thorough comprehension of the connection between alcohol consumption and RSB in the South African context [37]. The current study seeks to quantitatively investigate alcohol use, gender, and employment to gain a better understanding of the factors that predict RSB. Once we have a foundational level of understanding, we can then build on this model to test more sophisticated models.

### 1.2. Aim

The main aim of this study was to investigate the relation between risky drinking and RSB. This study further aimed to examine whether the severity of alcohol dependence (assessed using the AUDIT), gender and employment status predict RSB.

## 2. Methods

### 2.1. Study Design and Context

A cross-sectional correlational survey design was utilised in the study. The research was carried out in two low socioeconomic status communities in the Cape Flats in the Western Cape of South Africa. The Cape Flats is located 15 kilometres from Cape Town. It is a large flat area on the outskirts of Cape Town that includes “Coloured” and “Black” townships [50]. “Coloured” and “Black” were used as racial categories to enforce a racist ideology of segregation during apartheid. The apartheid era refers to the period when these population groups (including Indians) were oppressed, disenfranchised, and denied access to resources. Due to apartheid-era spatial planning, these townships are marked by material deprivation and poverty, high rates of unemployment, crime, and drug abuse, low levels of educational attainment, inadequate infrastructure, and basic services [51]. While these communities are characterised by inequalities, with limited resources and access to intervention and treatment for alcohol- or substance-related disorders, consumption levels remain high [52,53].

### 2.2. Sampling

The study sample consisted of 1001 participants (51% women) ranging in age from 18 to 25 years. This age group has been identified as having the highest prevalence of alcohol consumption in South Africa [46]. The total population size of each community is 6527 and 2234, respectively, for young adults [54,55]. The sample size was calculated by using a 99% confidence level, 5% margin of error, and the size of each community, which led to a recommended sample size of 655, while the final achieved sample size was 1001. Purposive sampling was used to select these participants, with age and place of residence serving as inclusion criteria. We employed the street-intercept method—a unique data collection strategy used to gather information in natural real-world environments, e.g., in public spaces such as suburban streets, parks and green areas, shopping centres, or other locations where people congregate. This method involves researchers directly approaching and engaging with people in these public settings. It allows researchers to capture spontaneous and unfiltered responses and behaviours, making it particularly useful for studying social and health-related human behaviours, such as alcohol use and sexual behaviour [37,56]. The street-intercept technique has been employed in ascertaining the prevalence of alcohol [56,57] and crystal methamphetamine use [58], and it is often used owing to its high level of feasibility. Its effectiveness is contingent on the rigorous application and adherence to best practice standards relating to sampling, site selection, training of data collectors, and application of key ethics principles. The sample recruitment process for street-intercept data collection necessitates a systematic approach, integral to the attainment of a diverse and representative participant pool. In the current study, the data were collected by the principal investigators, who all have substantial experience in working with youth and young people. We embarked on a meticulous process of site selection, which included a popular retail district and transport hub. These sites allowed us access to a large range of young people, encompassing a spectrum of characteristics and demographics. We approached potential participants with a precise introduction to the research project, its overarching goals, the ethics governing the study and their potential role in the process. The quality of our engagement played a pivotal role in fostering interest and willingness to participate. In the context of this study, the participation rate was empirically determined to be 60%, attesting to the efficaciousness of the data collection and recruitment process. This rate further exemplifies the resonance of the research within the community context and the collective commitment of participants to contribute to the study’s objectives.

### 2.3. Instruments

We used the Alcohol Use Disorder Identification Test (AUDIT) and the Self-Report Risky Sexual Behaviours Scale (SRSBS) [35] in this study. The scales were cognitively tested and adapted, in English and Afrikaans, with a South African sample of young adults from the target population. The questionnaire was available to the participants in Afrikaans or English, which are two of the most widely spoken languages in the province. The instruments were administered to the participants to examine risky drinking and its relation to RSB.

#### 2.3.1. Alcohol Use Disorder Identification Test (AUDIT)

The World Health Organization developed the AUDIT to assess excessive drinking and hazardous and harmful patterns of alcohol consumption [59]. In other words, the AUDIT measures the level of risky alcohol consumption, hereafter referred to as risky drinking. The scale has ten items, three of which assess drinking frequency and quantity, three of which assess alcohol dependence, and four of which assess alcohol-related complications. The items are administered and scored on a five-point Likert scale in approximately two minutes [60,61]. The possible composite scores range from 0 to 40, with 0 indicating someone who abstains from using alcohol [59]. According to the WHO guidelines, scores of 1–7 suggest low-risk consumption, 8–14 harmful consumption, and 15 upward a likelihood of dependence. The AUDIT has been shown to measure a single construct, i.e., the level of risky use of alcohol. The scale has displayed acceptable test—retest and internal consistency ranging from 0.76 [62] in Uganda to 0.83 [35,61,63] and 0.98 [64]. A recent South African study reported a Cronbach’s alpha of 0.89 [59]. Validation studies confirmed the content [64], criterion (predictive, concurrent), and construct validity [60,61].

#### 2.3.2. Self-Report Risky Sexual Behaviours Scale (SRSBS)

The 38-item SRSBS was used to evaluate sexual risk behaviours, such as self-reported unprotected sex, condom use, substance use related to sex, and sexual negotiations. The items of the scale are arranged on a dichotomous scale (0 = no, 1 = yes). Examples of the items are: “Have you had sex with someone you’ve known for less than a day?” and “Have you had sex without discussing condom use?” In addition, the reliability (Cronbach’s alpha) of the SRSBS was reported to be acceptable at 0.79 in a sample of college students [35].

Therefore, the above-mentioned instruments that were previously utilised were deemed fit for the current study as they have been used within the same context as the present study.

### 2.4. Procedure and Ethics

The University of the Western Cape Senate Research Committee granted the study ethics approval (registration no. 13/5/20). The study was conducted between 2018 and 2019. We used the street-intercept method to recruit participants. The study’s purpose, its intended role, and the fundamental ethical principles guiding the research were all explained to prospective participants by the researchers as they approached them in public areas of their communities. Due to the sensitive nature of the questions, anonymity and the ability to withdraw without consequences were guaranteed to participants. Participants gave written informed consent before completing the survey. Our data collection procedure included a referral mechanism, enabling participants’ access to free counselling services.

### 2.5. Data Analysis

The data were analysed via multivariate multiple regression analysis using Stata 14SE (Stata Corp.), (College Station, TX, USA). First, descriptive statistics were generated for the AUDIT and SRSBS. Second, we ran a simple regression to examine whether alcohol consumption was a significant predictor of RSB. Finally, to achieve the aims of the study, we used hierarchical multiple regression to examine whether alcohol consumption, gender, and employment status were significant predictors of RSB. Risky drinking (AUDIT categories: abstainer, low risk, harmful, and dependent), gender (man or woman) and unemployment status (employed or unemployed) were included as categorical predictors in the model, with RSB as the outcome variable.

The authors and research assistants captured the data, and the final dataset was cleaned, verified, and depurated by the principal investigators of the study. Missing data were imputed using regression in SPSS 25.

## 3. Results

### 3.1. Descriptive Statistics

Table 1, Table 2 and Table 3 show the descriptive statistics concerning the study variables. The skewness for the AUDIT ranged from 0.15 (How many alcoholic drinks do you have at a time when drinking?) to 1.08 (How often during the last year have you felt bad or guilty after drinking?), and the kurtosis ranged from 1.32 (Have you or someone else been injured because of your drinking?) to 3.02 (How often during the last year have you felt bad or guilty after drinking?). For the SRBS, RSB was continuous. The skewness ranged from 0.03 (With sexual partners, do you discuss practicing safe sex?) to 4.57 (Within the last month, have you had sex with an IV drug-using partner 3 or more times?), while the kurtosis ranged from 1.00 (With sexual partners, do you discuss practicing safe sex?) to 21.88 (Within the last month, have you had sex with an IV drug-using partner 3 or more?).

Table 1 presents the AUDIT scale categories and cut-off scores. In the current sample, nearly half of the participants (48.7%) were categorised as having “alcohol dependence”, which is concerning (487 participants out of 1001). Additionally, 23.1% of participants were classified as “harmful” drinkers, 25.6% as “low-risk” alcohol consumers, and 2.7% were “abstainers”.

Table 2 presents the mean score for the AUDIT. The table indicates that this sample obtained slightly high scores for consuming alcohol; therefore, risky drinking is high among this cohort (14.61). The average score of 14 on the AUDIT indicates that, on average, the sample demonstrates hazardous levels of alcohol consumption. This is in line with the statistics reported [36] and supports the rationale for conducting the study among young adults in a South African context.

Table 3 presents the mean score for the SRSBS. The table indicates this sample obtained slightly high scores, demonstrating a possible tendency to engage in RSB (12.78).

The following table shows the results of a simple linear regression of risky drinking as a predictor of RSB.

Table 4 presents the results of a simple regression to examine whether risky drinking [95% CI: 0.285, 0.358] is a predictor of RSB. We found the overall model to be significant (F = 296.50, *p* < 0.01). When risky drinking is increased by one unit, the regression model predicts that an increase of 0.481 will occur in RSB. The model indicates that risky drinking explains 23.22% of the variance in RSB (R^2^ = 0.23).

Multiple regression analysis was used to develop a model for predicting if risky drinking, gender, and employment status were predictors of RSB (see Table 5).

Table 5 presents the results of a multiple regression to examine whether risky drinking [95% CI: 0.28, 0.35], gender [95% CI: −1.69, −0.22] and employment status [95% CI: −0.89, 0.62] are significant predictors of RSB.

We found the overall model to be significant (F = 50.28, *p* < 0.001). Risky drinking (*p* < 0.001) and gender (*p* < 0.0.1) were found to be significant predictors of RSB, but not employment status (*p* > 0.05). More specifically, when considering the categories of risky drinking (low risk, harmful, and dependence), in comparison to the base group, low-risk drinking increased RSB by 4.08, harmful drinking increased RSB by 6.12, and alcohol dependence increased RSB by a substantial 9.95. Being a woman, in comparison to the base group of men, decreased RSB by −1.03. The model indicates that risky drinking and gender explain 18.41% of the variance in RSB (R^2^ = 0.1841). This is a slight decrease from the simple linear regression (risky drinking and RSB) of 22%.

We ran a model adding risky drinking (as a composite score) as a quadratic term and found that it was a non-linear variable, demonstrating a positive curvilinear relationship. We also ran the predictive margins and found that the expected value for RSB for this model is 12.78 (95% CI: 12.41–13.14).

### 3.2. Post Hoc Tests

We ran post hoc analyses (Bonferroni comparison) on the regression model to determine whether significant differences existed between RSB and alcohol consumption across gender (man or woman) and employment status (unemployed or employed). We included gender and employment status separately and as an interaction term (gender*employment status). We found that risky drinking for those unemployed was not significant across men and women, while for those employed, it was significantly higher for women (*p* < 0.001). Finally, RSB was significant across gender for those unemployed (higher for men) and for those employed (higher for men).

## 4. Discussion

Risky drinking was found to be a significant predictor of RSB. This finding adds to a growing body of research indicating a strong link between alcohol and sexual risk behaviour [19], especially when drinking occurs during sexual events [5,65]. The use of alcohol explains 23.22% of the variance in RSB. Furthermore, risky drinking and gender were found to be significant predictors of RSB, but not employment status. However, here it is important to consider the three categories of risky drinking using the AUDIT [59], namely low-risk, harmful, and dependent drinking, which increased RSB. Low-risk drinking is defined as participants scoring below the cut-off scores, harmful drinking scoring above the cut-off scores, who most likely would benefit from a brief intervention, and dependent drinking scoring very high, who most likely should be referred for diagnostic evaluation and more intensive treatment [59]. Essentially, the study confirms that the degree of alcohol misuse predicts increased engagement in RSB, which escalates per category. In addition, risky drinking and gender explain 18.41% of the variance in RSB, demonstrating good explanatory power (within social science research); this also enables researchers to consider other explanatory variables. Low-income communities in South Africa have disproportionately higher rates of substance use disorders as a result of structural and historical factors [66]. However, alcohol use is non-linear, as alcohol use increases from adolescence to young adulthood and then decreases after a certain age. As alcohol use increases, the likelihood of RSB increases. This includes sexual encounters without discussing the use of a condom, multiple sexual partners, and partners known for less than 24 h. Alcohol use is regarded as a significant risk factor for premature death and disease burden [67]. As a result, alcohol is a significant impediment to meeting 13 of the 17 SDGs by 2030.

In this sample, gender was identified as a significant predictor of RSB, with levels of RSB being higher in men than women. A number of explanations could potentially account for this; however, the most notable is the stigma attached to reporting sexual activity and alcohol use. According to South African women’s drinking patterns, 24% report alcohol use, and those who do drink tend to drink hazardously [65]. In South Africa, women’s drinking habits have remained consistent over time, with little evidence of a decline. While a recent study indicated that women are more inclined to engage in sexual activity than men without using a condom [68], this could be owing to a power disparity between men and women. This could influence women’s ability to choose to use a condom. Women were more likely to engage in sexual activity without a condom and for financial gain, while men were more likely to engage in such behaviour while intoxicated. However, women generally drink less than men [68]. Given that approximately 20% of women of childbearing age in South Africa are HIV-positive, the health consequences of alcohol use for women are severe. Additionally, South Africa has one of the highest incidences of gender-based violence (GBV) and alcohol consumption per capita in the world [69].

Employment status showed no significant contribution to RSB. However, we found that risky drinking among unemployed men and women was not significantly different, whereas it was significantly higher among employed women (*p* < 0.001). Furthermore, the group size for each category may have affected the result of unemployment. This could be a result of the communities in which the data were collected, which are characterised by inequalities, limited resources [52], and a high unemployment rate [43]. While the street-intercept method is useful within public health and development studies when traditional survey methods fall short or one is unable to obtain a representative sample, the sensitivity of the questionnaire and the environment in which data are collected can also impact the results [70]. In addition, previous research has linked hazardous drinking to a range of risk factors, including gender, age, socioeconomic factors such as parental income and level of education, and lifestyle [71]. The dynamics around exploring employment status as a contributing factor to alcohol consumption represent a complex issue that requires further exploration in the South African context, particularly since unemployment rates are so high amongst young adults [72,73] and more so because risk behaviour is prevalent amongst unemployed youth [74]. Therefore, the impact of employment status requires further exploration, as income (including parental income or socioeconomic status) remains an important predictor of alcohol use [75]. The history of institutionalised racism, structural violence, and social oppression in South Africa, along with high levels of social inequality, poverty, and other social and health factors, all contribute to South Africa’s prevalence of alcohol consumption and RSB [76].

### Limitations of the Study

We note a few limitations of this study. First, data were collected from a purposive sample utilising the street-intercept method. Owing to the sensitive nature of alcohol use and RSB, there is stigma attached as a result of social norms or concerns about anonymity, which may have contributed to underreporting [70]. Additionally, our findings cannot be generalised to other populations. Second, given the focus of the study, there may be other variables that could potentially mediate or moderate the relation between risky drinking and RSB, which could be explored in further research.

## 5. Conclusions

The current research supports and adds to the expanding body of knowledge [5,6,7,8,30] finding a significant relation between risky drinking and RSB among young adults living in low-income communities in South Africa. The study investigated the relation between risky drinking and RSB among young adults. The study further examined whether the severity of alcohol use, gender and employment status predicted RSB. Despite the study finding that employment status was not a significant contributor to RSB, the environment in which the data were collected needs to be considered, as research indicates high levels of unemployment at these research sites. Therefore, future research should consider conducting research across socioeconomic status groups to ensure a range of employment statuses are included. This research could consider using more sophisticated designs and multiple modes of data collection to be able to determine the direction of the relation of potential moderating variables. Furthermore, the study’s findings reveal the need for a better understanding of gender as a contributor to the relation between risky drinking and RSB. Therefore, a proportionate national representative sample would also benefit future research, including longitudinal research to determine patterns of risky drinking in the general population (including young adults).

## Figures and Tables

**Table 1:** Frequency of AUDIT categories.

AUDIT Categories	Cut Score	Frequency	Valid %
Abstainer	0	27	2.7
Low risk	1–7	256	25.6
Harmful	8–14	231	23.1
Alcohol dependence	15–40	487	48.7
TOTAL	–	1001	100

**Table 2:** AUDIT mean score.

	Mean	Std. Err.	[95% Conf. Interval]	
AUDIT	14.61	0.32	13.99	15.24

Number of obs. = 1001.

**Table 3:** SRSBS total mean score.

	Mean	Std. Dev.
RSB	12.78	6.79

**Table 4:** Simple regression output: RSB and risky drinking.

RSB	Coef.	Robust Std. Err.	*t*	*p* > |*t*|	[95% Conf. Interval]	
Risky drinking	0.32	0.019	17.22	0.001	0.29	0.36
Constant	8.07	0.37	21.97	0.001	7.35	8.79

Number of obs. = 1001; F (1, 999) = 296.50; Prob > F < 0.001; R-squared = 0.23; Root MSE = 5.95.

**Table 5:** Multiple regression: Risky drinking, gender, and employment status as predictors of RSB.

RSB	Coef.	Robust Std. Err.	T	*p* > |*t*|	[95% Conf. Interval]	
Risky drinking categories						
Low risk	4.08	0.98	4.17	0.001	2.16	5.99
Harmful	6.12	0.91	6.76	0.001	4.35	7.90
Dependence	9.95	0.89	11.22	0.001	8.21	11.69
Gender						
Female	−1.03	0.39	−2.66	0.010	−1.78	−0.27
Employment status						
Employed	−0.70	0.39	−1.79	0.070	−1.48	0.07
Cons	6.35	0.93	6.87	0.001	4.54	8.17

Linear regression; Number of obs. = 1001; F(3, 997) = 50.28; Prob > F < 0.001; R-squared = 0.18; Root MSE = 6.14. Note: The base category for alcohol consumption was abstainer (abstainer = 0; low risk = 1; harmful = 2; alcohol dependence = 3); for gender, it was men (men = 0; women = 1); and for employment status, it was unemployed (unemployed = 0; employed = 1).

## Data Availability

The data presented in this study are available on request from the corresponding author.
